# Profiling ribonucleotide and deoxyribonucleotide pools perturbed by gemcitabine in human non-small cell lung cancer cells

**DOI:** 10.1038/srep37250

**Published:** 2016-11-15

**Authors:** Jian-Ru Guo, Qian-Qian Chen, Christopher Wai Kei Lam, Cai-Yun Wang, Vincent Kam Wai Wong, Zee-Fen Chang, Wei Zhang

**Affiliations:** 1 State Key Laboratory of Quality Research in Chinese Medicine, Macau Institute for Applied Research in Medicine and Health, Macau University of Science and Technology, Taipa, Macau, China; 2 Institute of Molecular Medicine; College of Medicine; National Taiwan University, Taipei, Taiwan (R.O.C.)

## Abstract

In this study, we investigated the dosage effect of gemcitabine, an inhibitor of ribonucleotide reductase (RR), on cellular levels of ribonucleotides and deoxyribonucleotides using high performance liquid chromatography-electrospray ionization tandem mass spectrometric method. As anticipated, after 4-h incubation of non-small cell lung cancer (A549) cells with gemcitabine at 0.5 and 2 μM, there were consistent reductions in levels of deoxyribonucleoside diphosphates (dNDP) and their corresponding deoxyribonucleoside triphosphates (dNTP). However, after 24-h exposure to 0.5 μM gemcitabine, the amounts of dNTP were increased by about 3 fold, whereas cells after 24-h 2 μM gemcitabine treatment exhibited deoxycytidine diphosphate (dCDP), deoxyadenosine diphosphate (dADP) and deoxyguanosine diphosphate (dGDP) levels less than 50% of control values, with deoxycytidine triphosphate (dCTP) and deoxyguanosine triphosphate (dGTP) returning to the control level. Using cell cycle analysis, we found that 24-h incubation at 0.5 μM gemcitabine resulted in a significant increase in S phase arrest, while 2 μM treatment increased G0/G1 population. Our data demonstrated the correlation between the level of RR and the increased levels of dNTPs in the group of 0.5 μM treatment for 24-h with a markedly reduced level of dFdCTP. Accordingly, we proposed that the dosage of dFdC could determine the arrested phase of cell cycle, in turn affecting the recovery of dNTPs pools.

Gemcitabine (2′,2′-difluroro-2′-deoxycytidine; dFdC) is a deoxycytidine analogue for chemotherapy of lung cancer and other solid tumors^1^,^2^,^3^. It is a prodrug which requires intracellular metabolism by nucleoside kinases to its active metabolites including gemcitabine diphosphate (dFdCDP) and gemcitabine triphosphate (dFdCTP)^4^,^5^,^6^. Gemcitabine exerts its cytotoxic effect mainly through active dFdCTP that competes with deoxycytidine triphosphate (dCTP) for incorporation into DNA and leads to inhibition of DNA synthesis. On the other hand, dFdCDP can inhibit ribonucleotide reductase (RR), which is a key enzyme catalyzing the formation of deoxyribonucleotides (dRN) from ribonucleotides (RN)^7^,^8^,^9^. Inhibition of RR decreases the deoxynucleotide pool sizes for DNA repair and synthesis. The reduction in the intracellular concentration of dCTP caused by the inhibition of RR will also help the incorporation of dFdCTP into DNA. This is a unique mechanism of gemcitabine known as ‘self-potentiation’^10^.

It is well known that the action of dFdC against cancer can affect endogenous RN and dRN pool sizes that play essential roles in a broad range of key cellular functions. Unbalanced change of deoxyribonucleoside triphosphates (dNTP) caused by the dFdC or other nucleotide analogues can lead to genetic abnormalities or cell death in mammalian cells^11^. The action of nucleoside analogues against cancer and viral infection can also be affected by RN and dRN pool sizes^12^,^13^. In order to understand the exact mechanism of action of dFdC, it is critical to elucidate the disturbances of dFdC treatment on RN and dRN pool sizes since this may play an important role in its side effects or drug resistance.

Peters *et al.* have reported previously different effects of dFdC on ribonucleoside triphosphates (NTP) in twenty-one solid tumour and leukaemia cell lines. After treatment of dFdC, cytidine triphosphate (CTP) pool was increased about 2-fold in 12 out of 21 cancer cell lines, while 1.6–1.9 fold increases in adenosine triphosphate (ATP), uridine triphosphate (UTP) and guanosine triphosphate (GTP) pools were observed in 19–20 cell lines^14^. It has also been reported that dFdC caused a significant depletion of cellular dNTP with the most pronounced reduction in the dCTP pool^15^,^16^,^17^. Despite of these previous studies, little information regarding the alteration in monophosphate (dNMP) and diphosphate deoxyribonucleotides (dNDP) is available, because their amounts are much lower than the respective triphosphate metabolites. We have previously developed a HPLC/MS/MS method to study the perturbation of RN and dRN in cancer cell lines incubated with hydroxyurea, aphidicolin and 5-fluorouracil^18^,^19^. Using our method, intracellular metabolites of dFdC including gemcitabine monophosphate (dFdCMP), dFdCDP and dFdCTP can be measured simultaneously in a single analysis. Considering the importance of dFdC as the most effective agents for treating early and advanced stage NSCLC during the last twenty years^20^,^21^,^22^, a major goal of this study was to investigate the interaction of dRN and dFdC intracellular metabolites in non-small cell lung cancer (NSCLC) cells upon treatment with gemcitabine, The information obtained from this study should facilitate animal experiments and clinical trials to assess the efficacy and toxicity of dFdC for developing the individualized chemotherapy.

## Results

### Multivariate statistical analysis

**Figure 1:**
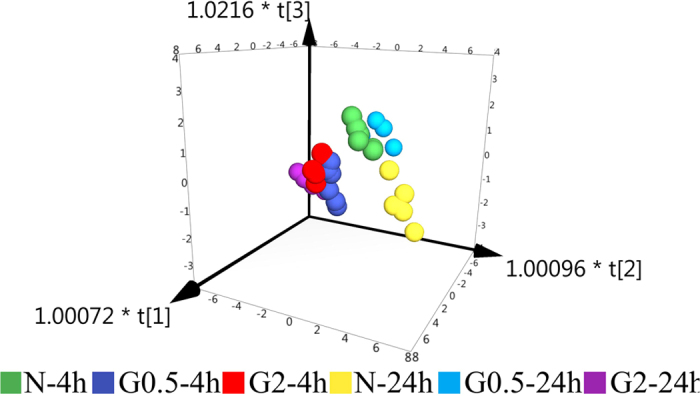
Scores plots of OPLS-DA models (N-4 h: Normal control group at 4 hour, G0.5–4 h: 4 h exposure with dFdC at 0.5 μM, G2–4 h: 4 h exposure with dFdC at 2 μM, N-24 h: Normal control group at 24 h, G0.5–24 h: 24 h exposure with dFdC at 0.5 μM, G2–24 h: 24 h exposure with dFdC at 2 μM).

Absolute amount of each deoxyribonucleotides and ribonucleotides was used to obtain a data matrix consisting of 36 objects and 24 variables. In order to understand and visualize the grouping trends in samples treated with dFdC at different doses and time periods, orthogonal partial least squares discriminant analysis (OPLS-DA) was used to assess the similarity of the different groups. In OPLS-DA analysis, the value of R2Y has been calculated to represent fraction of cumulative Y variance by a specific model component. At the same time, the value of Q2 predicts the accuracy of the model according to cross validation. Usually models with Q2 values greater than or equal to 0.5 indicate good predictive capability^23^,^24^. The OPLS-DA scores plot in Fig. 1 shows a significant separation before and after treatment of A549 cells with dFdC. Samples from the control group at different times and the treatment with dFdC at 0.5 μM for 24 h can be considered as one cluster. The others samples treated with dFdC at 0.5 μM for 4 h and 2 μM for 4 h and 24 h can be divided into other clusters. This indicates that A549 cells began to exhibit some extent of repair after 24 hour exposure to dFdC at 0.5 μM. Furthermore, the values of R2Y and Q2 for the OPLS-DA model were calculated as 0.709 and 0.50, respectively. These results indicate that dFdC incubation is associated with changes in RN and dRN pool sizes.

### The cell cycle alteration by dFdC treatment in A549 cells

**Figure 2:**
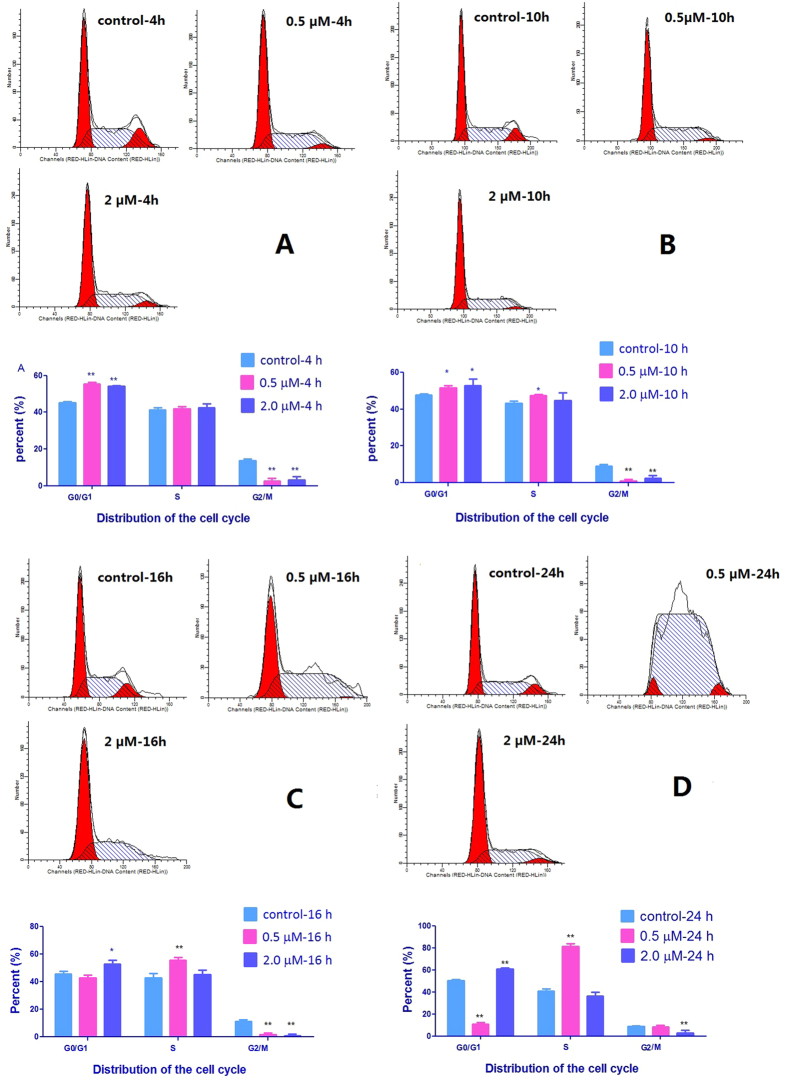
Effects of dFdC on cell cycle arrest in A549 cells at different times. (**A**) 4 h; (**B**) 10 h; (**C**) 16 h; (**D**) 24 h. *P*-value of less than 0.05 (*P < 0.05, **P < 0.01, compared with the control group) are considered significant.

The cell cycle is a cyclic process of cell division. dFdCTP can incorporate into DNA to inhibit DNA synthesis. Treatments of A549 non-small cell lung cancer (NSCLC) cells with dFdC at 0.5 and 2.0 μM for 4 h resulted in similar increases in percentage of cells at G0/G1 phase from 45.1 ± 0.5 to 55.4 ± 0.7 and 54.2 ± 0.2%, respectively, both P < 0.01 (Fig. 2). However, a significant difference between the two doses of dFdC was observed in the profile of cell cycle after 10 h treatment. After treatment with dFdC at 0.5 μM, there was a progressive increase in S phase arrest from 10 to 24 h. Finally, treatment with dFdC at 0.5 μM for 24 h induced cell cycle arrest at S phase from 40.9 ± 1.5 to 79.6 ± 0.1% (P < 0.01). In contrast, the percentage of G0/G1 phase cells was increased from 50.3 ± 0.9 to 61.4 ± 0.8% in the group of 24-h treatment with dFdC at 2 μM (P < 0.01). Thus, dFdC at lower concentration induces cell cycle arrest in the S phase, whereas higher concentration induces arrest in G0/G1 phase.

### Perturbation of RN pool size by dFdC in A549 cells

Tables 1 and 2 show the general properties and differences in RN and dRN pool sizes of A549 cells before and after dFdC treatment. The energy charge of A549 cells, which is defined as (ATP + 0.5 ADP)/(ATP + ADP + AMP), changed from 0.92 to 0.96 before and after treatment. After 4 h of incubation, there was no significant difference in energy charge with or without dFdC. At the same time, we did not observe any significant changes in ATP levels. There were significant increases in ATP/dATP, CTP/dCTP and GTP/dGTP ratios due to the decrease of corresponding dNTP. However, after 24 h incubation, the amount of ATP increased from 15767.40 ± 1047.07 to 38050.63 ± 4295.90 pmol/10^6^ cell at 0.5 μM dFdC and to 45581.84 ± 5391.24 pmol/10^6^ cell at 2 μM dFdC, respectively. In addition, CTP, GTP and UTP demonstrated similar increases at two concentrations of dFdC (Table 1). The effects of dFdC on ribonucleotide triphosphates have already been reported and possibly related with the cytostatic effect of gemcitabine^25^,^26^. Of note, the inhibition of ribonucleotide reductase by dFdC after 24 h of incubation did not significantly increase the levels of ribonucleotide diphosphates, except for adenosine diphosphate (ADP). This is probably because the amount of ribonucleotide diphosphates were almost a hundred times greater than deoxyribonucleotide diphosphates in the cellular pool sizes.

### Perturbation of dRN pool size by dFdC in A549 cells

**Figure 3:**
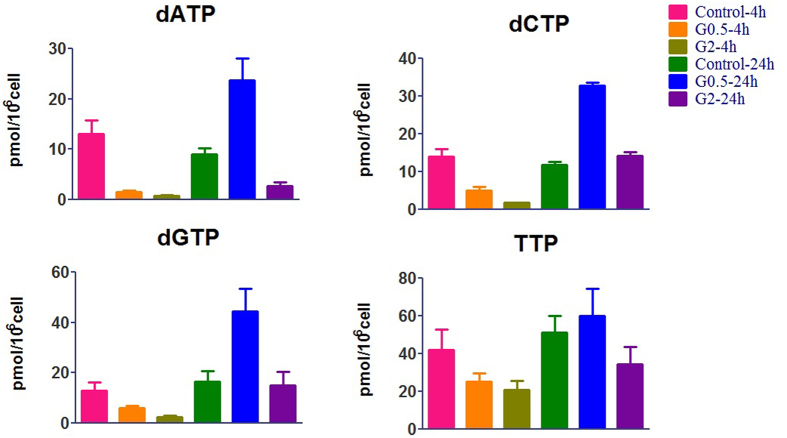
Effects of dFdC on dNTP pool sizes after treatment of A549 cells with 0.5 and 2.0 μM of dFdC. Each data point is an average of two independent experiments (done in triplicate) and is reported as mean ± standard deviation values.

**Figure 4:**
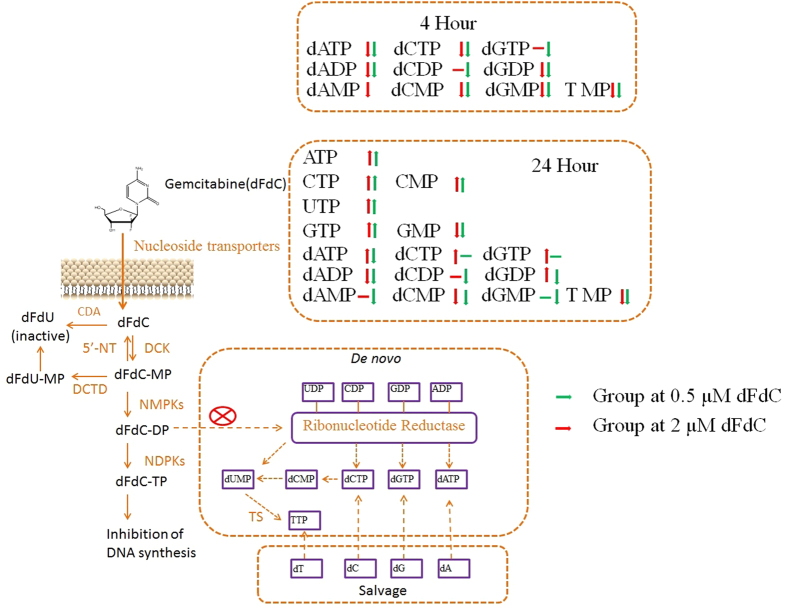
Summary of the metabolites of dFdC and its major metabolic pathways as well as major perturbations of RN and dRN. NMPKs, Nucleoside monophosphate kinases; NDPKs, Nucleoside diphosphate kinases; CDA, cytidine deaminase; DCK, deoxycytidine kinase; 5′-NT, 5′-nucleotidase; DCTD, deoxycytidine monophosphate deaminase.

Table 2 and Fig. 3 summarize dRN levels in A549 cells before and after incubation with dFdC at different doses and time periods. In untreated cells, deoxythymidine triphosphate (TTP) was the biggest dNTP pool (34.89 ± 16.50 pmol/10^6^ cell) when compared with dATP (13.02 ± 6.07), dCTP (14.03 ± 4.55) and dGTP (12.84 ± 7.91). As expected, 4-hour exposure to 0.5 and 2 μM dFdC reduced cellular dCDP, dADP and dGDP to below 50% of control values because of inhibition of ribonucleotide reductase. The depleted (< 50% of control) dCDP, dADP and dGDP led to decreases in dCTP, dATP and dGTP, respectively. After 4-h exposure, the most pronounced decrease was in the dATP pool, followed by decreases in dCTP, dGTP, and dTTP at two dosages of dFdC. Regular supply of endogenous TMP is predominantly through dCMP and dUMP by dCMP deaminases and thymidylate synthase. Conceivably, the TMP pool depletion was caused by the decease of dCMP. Both two dosage groups showed significant differences after 24-h exposure. With the dose of 0.5 μM dFdC, the recovery of dNDP suggests that the loss of dFdC impact on the interference in deoxynucleotides supplies. In addition, the amount of dNTP increased about 3 fold after 24 h of 0.5 μM dFdC incubation. However, 2 μM dFdC should still inhibit ribonucleotide reductase after 24-h exposure because the concentrations of dCDP, dADP and dGDP were still less than 50% of control values. At the same time, there was similar dATP pool depletion, which is consistent with a previous study that gemcitabine produces a profound depletion of dATP^27^. Finally, major metabolic pathways of dFdC and its impact on RN and dRN (above/below 50% of control) are summarized in Fig. 4.

Under our experimental conditions, the accumulation of dFdCTP, determined at two concentrations (0.5 and 2 μM) were 14.20 ± 7.58 and 67.50 ± 18.52 pmol/10^6^ cell for 4-h exposure and 0.23 ± 0.12 and 8.95 ± 0.68 pmol/10^6^ cell for 24-h exposure, respectively. Maximal accumulation of dFdCTP was achieved after 4-h exposure to 2 μM dFdC. The levels of active metabolites of dFdC appeared to be dose-dependent. As anticipated, the dFdCTP level in A549 cells was significantly higher than that of dFdCDP and dFdCMP. The percentage changes in dNDP suggest that the inhibition of RR correlated with the increase of dFdCDP.

### Western blot analysis of cellular RR expression regulated by dFdC

**Figure 5:**
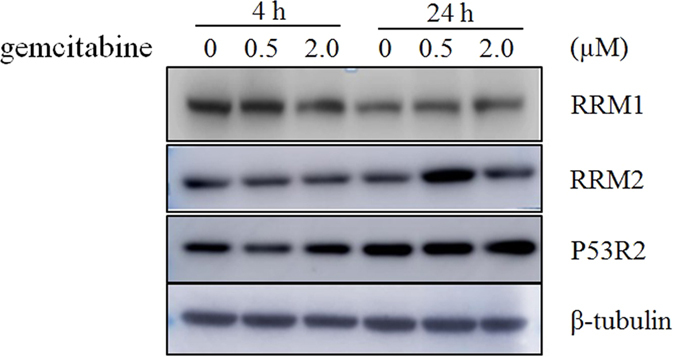
Western blot analysis of the effect of dFdC on expression of ribonucleotide reductase subunits M1 (RRM1) and M2 (RRM2), P53-controlled ribonucleotide reductase (P53R2).

Usually, the concentration of dNTP is highest in S phase and lowest in G1 phase, which is controlled by RR^28^. Human RR is composed of three known subunits, RRM1 (large subunit), RRM2 (small subunit) and an encoded P53-controlled ribonucleotide reductase (P53R2) that are differentially regulated during the cell cycle^29^,^30^. R1 protein concentrations are relatively constant throughout the cell cycle. M2 protein is low outside S phase. It has already been reported that RRM1 may be related to the gemcitabine response^31^,^32^. To further investigate these observations, we determined the expression of RRM1, RRM2 and P53R2 using the Western blot. After 4-h incubation, 2 μM dFdC slightly reduced RRM1 level, whereas no effects on RRM2 and P53R2 were observed (Fig. 5). The expression of RRM2 was significantly increased after 24-h incubation with dFdC at 0.5 μM, which was caused by cell cycle arrest at S phase. At the same time, high level of RRM2 expression at 0.5 μM dFdC was associated with high level of dNDPs. Although there were no changes in the levels of three subunits of RR after 24-h exposure to 2 μM dFdC, the concentrations of dCDP, dADP and dGDP were still less than 50% of control values. These findings suggest that dFdC may still have inhibition impact on RR.

## Discussion

In this study, individual levels of RN, dRN and active metabolites of dFdC have been simultaneously analyzed to elucidate the chemotherapeutic mechanism of dFdC. The potential differential responses of all RN and dRN to incubation with different concentrations and durations of dFdC were investigated. Generally, high levels of deoxyuridine pool sizes can lead to misincorporation of uracil into DNA, DNA double strand breakage, and induction of cell apoptosis. In this study, the levels of deoxyuridine triphosphate (dUTP), deoxyuridine diphosphate (dUDP) and deoxyuridine monophosphate (dUMP) were still under detection limit of the assay before and after treatment of dFdC. Thus deoxyuridine pool sizes are not shown in this manuscript.

Ribonucleotide monophosphates were not affected by 4-h exposure to dFdC. After 24-h incubation, 2 μM dFdC reduced cellular AMP and GMP, whereas both doses of dFdC increased CMP, but less than 1.5-fold. dFdCMP is phosphorylated to dFdCDP by UMP/CMP kinase (UMP/CMPK), which belongs to bifunctional nucleoside monophosphate kinases (NMPKs) family and plays an important role in phosphorylation of UMP, CMP, and dCMP^33^,^34^. Decreased levels of UMP/CMPK is an important mechanism of clinical resistance to fluoropyrimidine therapy^35^. Although there is no evidence to demonstrate the association of genetic polymorphisms or expression of UMP/CMPK with clinical outcome of dFdC, reduction of UMP/CMPK activity might lead to the decrease of active metabolites of dFdC and subsequently reduced cytotoxic effect. Therefore, it is possible that the increase of CMP is a result of inhibition of UMP/CMPK by dFdCMP. At present, it remains to be investigated why dFdC treatment decreases the levels of AMP and GMP.

Both 0.5 μM and 2 μM dFdC showed potent growth inhibitory effects on A549 cells. A549 cell number reduced from about 6.0 × 10^6^ cells to 2.7 × 10^6^ cells for 0.5 μM and 2.4 × 10^6^ cells 2 μM after 24-h exposure, respectively. As a consequence of DNA damage by dFdC, cell division is arrested in A549, which leads to changes in dNTP pool size. dFdC induces cell cycle arrest in the S phase at low concentration, whereas higher concentration induces arrest in the G0/G1 phase. Presumably, DNA damage can block cells to enter the S phase due to the presence of checkpoint control that inhibits the initiation of replication^36^,^37^. Our results indicate that the effect of dFdC on S phase arrest was inversely correlated with the levels of its active metabolites. Combining the results of OPLS-DA and RN and dRN pool sizes, the cells have significantly recovered after treatment with 0.5 μM dFdC for 24 h. Cancer cells could develop drug resistance through drug inactivation, drug target alteration, DNA mutations and metabolic changes. An increase in the expression of RRM2 may be highly correlated with drug resistant response of A549, which shows a similar mechanism for the development of resistance to methotrexate is amplification of the DHFR gene^38^.

In summary, the anti-tumor activity of gemcitabine (dFdC) is not only related to its active metabolites but also the marked changes in ribonucleotide (RN) and deoxyribonucleotide (dRN) pools caused by inhibition or stimulation of several important enzymes involved in RNA and DNA synthesis. The present study has elucidated the disturbance of RN and dRN pools, possibly providing an impact on antitumor response of NAs and cancer-cell selectivity. The observed alterations in pool sizes are consistent with the present understanding of actions of dFdC. The perturbation of dFdC on RN and dRN pools sizes may be equally important to its intracellular activities metabolites. Our study may help to understand how nucleoside analogues work and predict their clinical efficacy and toxicity.

## Materials and Methods

### Chemicals and reagents

LC-MS grade methanol, acetonitrile and acetic acid were purchased from Anaqua Chemical Supply Co., Houston, TX, USA. Gemcitabine, hexylamine (HA), diethylamine (DEA), trioctylamine, 1,1,2-trichlorotrifluoroethane, stable isotope labeled adenosine-^13^C_10_^15^N_5_-triphosphate (ATP^13^C^15^N), dimethyl sulfoxide (DMSO), trypsin-EDTA solution and 3-[(4, 5)-dimethylthiazol-2-yl]-2,5-diphenyl tetrazolium bromide (MTT) were purchased from Sigma Aldrich Chemical Co., St. Louis, MO, USA. Ultra-pure water was obtained from a Milli-Q Gradient Water System (Millipore Corp., Bedford, MA, USA). For culturing cells, phosphate buffered saline, pH 7.8 (PBS), Dulbecco’s Modified Eagle Medium (DMEM), penicillin–streptomycin solution and fetal bovine serum (FBS) were obtained from Gibco Invitrogen Corp., Carlsbad, CA, USA. Human NSCLC cell line (A549) was supplied by American Type Culture Collection (ATCC), Rockville, MD, USA. dFdCMP, dFdCDP and dFdCTP were synthesized chemically according to an established method^39^. dFdCMP, dFdCDP and dFdCTP were confirmed by HPLC (Shimadzu Scientific Instruments, Braintree, MA, USA) using a binary gradient of water and 0.3 M potassium phosphate buffer in an anion exchange column (Partisil-SAX, Whatman, Inc., Clifton, NJ, USA). Their concentrations were determined from the absorbance at 272 nm.

### LC/MS/MS Assay

This was performed on a Thermo Fisher TSQ LC–MS/MS system consisted of an Accela Autosampler, an Accela pump and a Quantum Access triple quadrupole mass spectrometer (Thermo Fisher Scientific Co., San Jose, CA, USA). Data acquisition was performed with the Xcalibur software version 2.0.7, and data processing using the Thermo LCquan 2.5.6 data analysis program (Thermo Fischer). The chromatographic separation was achieved using an XTerra-MS C_18_ column (150 mm X 2.1 mm i.d., 3.5 μm, Waters Corp., Milford, MA, USA). The two eluents were: (A) 5 mM HA–0.5% DEA in water, pH adjusted to 10 with acetic acid; and (B) 50% acetonitrile in water. The mobile phase consisted of a linear gradient of A and B: 0–15 min, 100–80% A (v/v); 15–35 min, 80–70% A; 35–45 min, 70–45% A; 45–46 min, 45–0% A; 46–50 min, 0–0% A; 51–70 min, 100–100% A. The liquid flow-rate was set at 0.3 mL/min, and the column temperature was maintained at 35 °C. For all RN and dRN, the following optimized parameters were obtained. The sheath gas pressure reached 40 psi. The ionspray voltage was set at 3000 V for negative mode and 4000 V for positive mode at a temperature of 350 °C and auxiliary gas pressure of 15 psi. Quantification was performed using multiple reactions monitoring (MRM) as previously published^18^.

### Cell Culture

The human A549 NSCLC cells were cultured in RPMI 1640 medium containing 10% (v/v) fetal bovine serum (FBS), 100 units/mL penicillin, 100 μg/mL streptomycin in a 37 °C humidified incubator with a 5% CO2 atmosphere. A549 cells were seeded in 100 mm X 20 mm dishes (Corning Inc, Corning, NY, USA). After overnight culture, cells were divided into two groups: control group and experimental group. Cells of the experimental group were incubated with 0.5 and 2 μM (ID_50_) of gemcitabine for 4 and 24 h. An extra dish of cell line was incubated for cell counting on the day of cell harvest for normalization of nucleotide pools, and cell viability was assessed by trypan blue exclusion assay (only cells with more than 95% viability were assessed). Control cells were incubated in medium only.

### Preparation of cell pellets

Monolayer A549 cells were washed with ice-cold PBS once and were trypsinized with 0.25% trypsin-EDTA. Cells from two or three dishes were then re-suspended with 12 mL ice-cold PBS. After centrifugation at 1,000 rpm for 5 min, the cell pellet was washed with 1 mL ice-cold PBS again and spun down at 1,000 rpm for 5 min. The cell pellet was incubated with 150 μL of 15% trichloroacetic acid (TCA) containing 7.5 μL of 20.0 μM ATP^13^C ^15^N as internal standard and placed on ice for 10 min. After centrifugation at 13,500 rpm for 15 min in the cold room, the acidic supernatant was separated and neutralized twice with 80 μL mixture of trioctylamine and 1,1,2-trichlorotrifluoroethane (45:55 v/v). Samples were stored at −80 °C until analysis within two days.

### Cell cycle analysis

Cells were seeded at 4.5 × 105 cells/well in 6-well culture plates in duplicate, and incubated with dFdC at 0.5 and 2 μM for 4 and 24 h. They were then harvested and fixed in 70% (v/v) cold ethanol overnight at 4 °C. The fixed cells were collected by centrifugation and re-suspended in PBS and incubated with 5 mg/mL propidium iodide (Sigma-Aldrich) and 10 mg/mL RNase A (Sigma-Aldrich) at room temperature for 30 min in the dark. The cells were then analyzed on a flow cytometer (MuseTM cell analyzer, Merck Millipore, Darmstadt, Germany). Finally, the percentages of cells in different phases (G0/G1, S and G2/M) were calculated using Modfit software (Verity Software House, USA)

### Western Blot analysis

All groups of cells were harvested and lysed in RIPA buffer (Cell Signaling Technologies Inc. Beverly, MA, USA). Bradford reagent (Bio-Rad Laboratories Inc, Hercules, CA, USA) was used to determine protein concentration. Cell lysates were mixed with 5ХSDS-loading buffer (4:1, v/v) and heated the tubes at 100 °C with locked capping for 5 min. Samples (40 μg of protein) were subjected to 10% SDS-PAGE. After electrophoresis, the cell extracts from SDS-PAGE were transferred to polyvinylidene fluoride transfer membrane (Bio Trace^TM^ PVDF, 0.45 μM, Pall Life Sciences Corp., Mexico). Membranes were then incubated with anti-RRM1 (D12F12) antibody (Cell Signaling Technology, Inc., MA, USA), anti-RRM2 (EPR11820) antibody, anti-p53R2 (EPR8816) antibody (Abcam Ltd, Cambridge, UK) and β-tubulin antibody (Santa Cruz Biotechnology, CA, USA) overnight at 4 °C, respectively. The membranes were further incubated with HRP-conjugated antibodies for one hour. The protein bands were visualized using the enhanced chemiluminescence reagents (Invitrogen Corp., Paisley, Scotland, UK), and were analyzed with the Image J 1.46r software (National Institutes of Health, Bethesda, MD, USA).

## Additional Information

**How to cite this article**: Guo, J.-R. *et al.* Profiling ribonucleotide and deoxyribonucleotide pools perturbed by gemcitabine in human non-small cell lung cancer cells. *Sci. Rep.* **6**, 37250; doi: 10.1038/srep37250 (2016).

**Publisher’s note:** Springer Nature remains neutral with regard to jurisdictional claims in published maps and institutional affiliations.

## Figures and Tables

**Table 1:** Levels of RN and general properties in A549 cells before and after incubation with dFdC at different time (pmol/10^6^ cell).

	control-4 h	0.5 μM-4 h	2.0 μM-4 h	control-24 h	0.5 μM-24 h	2.0 μM-24 h
ATP	17557.4 ± 413.5	19011.5 ± 2267.17	21151.8 ± 2973.0^*^	15767.4 ± 1047.1	38050.6 ± 4295.9^**^	45581.8 ± 5391.2^**^
ADP	1902.2 ± 471.3	2283.9 ± 470.23	2049.8 ± 385.4	2194.0 ± 341.6	3177.9 ± 767.7^*^	3098.0 ± 534.1^**^
AMP	197.6 ± 66.1	237.3 ± 112.20	177.4 ± 59.37	294.3 ± 53.9	239.5 ± 102.5	183.9 ± 22.2^**^
CTP	1608.1 ± 377.7	2001.9 ± 136.63*	2016.8 ± 171.0^*^	1422.4 ± 117.2	3611.7 ± 263.2^**^	4808.9 ± 382.2^**^
CDP	296.9 ± 111.9	399.6 ± 122.47	315.5 ± 106.5	368.9 ± 66.7	462.8 ± 160.2	453.1 ± 129.8
CMP	67.07 ± 25.87	82.11 ± 31.94	72.48 ± 21.27	86.30 ± 21.86	131.2 ± 30.00^*^	128.4 ± 27.14^*^
GTP	4638.1 ± 1161.8	5349.6 ± 833.83	5497.3 ± 625.1	4282.4 ± 1163.5	9595.8 ± 1601.7^**^	10439.3 ± 3063.4^**^
GDP	659.3 ± 122.1	801.9 ± 133.63	743.2 ± 112.1	859.0 ± 123.1	1096.7 ± 312.9	1024.1 ± 279.8
GMP	26.21 ± 12.37	31.36 ± 17.26	21.77 ± 10.33	49.48 ± 10.99	32.12 ± 18.78	17.69 ± 4.49^**^
UTP	9063.3 ± 711.9	10276.1 ± 746.54^*^	12060.3 ± 1231.1^**^	7523.2 ± 1007.1	18458.5 ± 3339.7^**^	23170.2 ± 3020.3^**^
UDP	794.2 ± 78.5	898.5 ± 165.67	811.9 ± 131.2	951.1 ± 173.8	985.6 ± 233.0	915.4 ± 87.6
UMP	25.26 ± 6.45	22.45 ± 4.31	23.30 ± 4.69	34.96 ± 14.76	23.38 ± 3.79	22.77 ± 10.07
ATP/dATP	1721.5 ± 805.1	18622.0 ± 12721.3^**^	35650.3 ± 19962.2^**^	1932.9 ± 594.6	1998.8 ± 984.9	32918.4 ± 25377.6^*^
CTP/dCTP	118.4 ± 12.2	482.5 ± 199.7^**^	1208.4 ± 201.6^**^	120.6 ± 10.2	110.6 ± 12.5	345.0 ± 51.5^**^
GTP/dGTP	536.7 ± 334.2	1045.9 ± 438.4^*^	2771.2 ± 1364.9^**^	364.4 ± 213.2	260.9 ± 99.1	1681.9 ± 1426.7^*^
Energy charge	0.94 ± 0.01	0.93 ± 0.02	0.95 ± 0.02	0.92 ± 0.01	0.95 ± 0.02^**^	0.96 ± 0.01^**^

Note: Each data point is an average of two independent experiments (each performed in triplicate) and is reported as mean ± standard deviation values. (*P < 0.05, **P < 0.01, compared with the control group).

**Table 2:** Levels of dRN in A549 cells before and after incubation with dFdC (pmol/10^6^cell).

	control-4 h	0.5 μM-4 h	2.0 μM-4 h	control-24 h	0.5 μM-24 h	2.0 μM-24 h
dATP	13.02 ± 6.07	1.53 ± 0.79**	0.80 ± 0.39^**^	9.00 ± 2.80	23.74 ± 9.58^**^	2.67 ± 1.78^**^
dADP	0.1324 ± 0.10	0	0	0.2248 ± 0.17	0.1448 ± 0.14	0
dAMP	0.0894 ± 0.049	0	0	0.1418 ± 0.086	0.1151 ± 0.086	0.0452 ± 0.050
dCTP	14.03 ± 4.55	5.04 ± 2.20^**^	1.74 ± 0.42^**^	11.90 ± 1.63	32.83 ± 1.88^**^	14.23 ± 2.20^*^
dCDP	0.1064 ± 0.052	0.0787 ± 0.056	0	0.2536 ± 0.159	0.2328 ± 0.17	0
dCMP	0	0	0	0.1764 ± 0.10	0	0
dGTP	12.84 ± 7.91	5.93 ± 2.19^*^	2.54 ± 1.35^**^	16.57 ± 9.17	44.37 ± 20.58^*^	15.09 ± 12.20
dGDP	0.67 ± 0.21	0.13 ± 0.03^**^	0	0.98 ± 0.34	1.54 ± 0.22^**^	0.19 ± 0.080^**^
dGMP	0.42 ± 0.35	0.12 ± 0.12^*^	0	1.08 ± 0.69	1.22 ± 1.10	0.14 ± 0.13^**^
TTP	34.98 ± 16.50	22.81 ± 6.67	19.48 ± 6.47^*^	31.94 ± 8.83	79.20 ± 25.14^**^	49.77 ± 19.45^*^
TDP	11.2 ± 1.63	9.68 ± 1.02^**^	7.46 ± 2.57^**^	14.54 ± 2.49	11.65 ± 2.69^**^	8.21 ± 1.84^*^
TMP	0.0467 ± 0.014	0.0166 ± 0.015^**^	0	0.1374 ± 0.044	0.0606 ± 0.061	0
dFdCMP	0	0.1 ± 0.02	0.74 ± 0.19	0	0	0
dFdCDP	0	0	0.32 ± 0.09	0	0	0
dFdCTP	0	14.2 ± 7.58	67.5 ± 18.52	0	0.23 ± 0.12	8.95 ± 0.68

Note: Each data point is an average of two independent experiments (each performed in triplicate) and is reported as mean ± standard deviation values. (*P < 0.05, **P < 0.01, compared with the control group).
